# 

**Published:** 1900-12-22

**Authors:** 


**IMPORTANT NOTICE.**

**On account of the Christmas Holidays, we beg to announce that we shall go to press with our issue of the 29th DECEMBER on MONDAY NIGHT, the 24th inst. Advertisements intended for insertion in this issue should therefore reach us by 4 p.m. ON MONDAY, the 24th inst.**

### NOTICE TO CORRESPONDENTS.

All MSS., Letters, Books for Review, and other matters intended for the Editor should be addressed The Editor, "The Hospital," The Hospital Building, 28 & 29 Southampton Street, Strand, W.C.

The Editor cannot undertake to return rejected MS., even when accompanied by stamped directed envelope.

**Notice to Advertisers and Subscribers.**

All Advertisements, Orders for Copies of The Hospital, and Business Communications should be addressed to **THE MANAGER (Not to the Editor),** The Hospital, 28 & 29 Southampton Street, Strand, London, W.C.

The Cost of Subscription, post free, is as follows:—Per week, 2½d.; per quarter, 2s. 8d.; per half-year, 5s. 3d.; per annum, 10s. 6d. Foreign:—Per annum, 15s. 2d., payable, in all cases, in advance.

**NOTICE.—Vol. XXVIII. of "The Hospital" (April to September, 1900), in handsome cloth binding, is now on sale at the Office of this Journal, price, 7s. 6d. Cases for binding the Numbers contained in this Volume 1s. 6d. Index to Vol. XXVIII., 2½d., post free.**

**The Monthly Part for January will be ready on January 1st. Price 6d., by post 8d.**

### The Student of Medicine.

### APPOINTMENTS.

**Daniel Elie Anderson,** B.A., B.Sc., M.B.Lond., M.R.C.S. Eng., L.R.C.P.Lond., L.S.A., M.D.Paris, appointed Visiting Physician to the Hertford British Hospital, Paris. **T. R. Atkinson,** M.D.Durh., appointed Medical Officer for the Third District of the Romford Union. **L. Bathurst,** M.R.C.S., L.R.C.P.Lond., appointed District Medical Officer of the Ellesmere Union. **G. Bernard,** M.D.Kingston, L.R.C.P., L.R.C.S.Edin., appointed District Medical Officer of the Sunderland Union. **G. H. Brand,** M.D.Brux., L.R.C.P., L.M.Irel., L.S.A.Lond., appointed Medical Officer for the examination of candidates for the Royal Navy and Royal Marines for the Northampton District. **J. Walter Brown,** B.A., M.B., B.Ch.R.U.I., appointed House-Surgeon to the Children's Hospital, Forest House, Nottingham. **W. Byford,** M.R.C.S., L.R.C.P.Lond., appointed Medical Officer of Health for the Borough of Ruthin. **R. Byrne,** B.A.R.U.I., L.R.C.P., L.R.C.S.Edin., appointed Extern House-Surgeon to the North Charitable Infirmary, Cork. **R. P. Cooke,** L.R.C.P., L.R.C.S.Edin., appointed Medical Officer for the Oldbury District of the West Bromwich Union. **Douglas Drew,** B.S., M.D.Lond., F.R.C.S.Eng., appointed Surgeon to the In-patients at the Hospital for Women, Soho Square. **R. Sidney Ellis,** M.B., Ch.B.Edin., appointed Resident Medical Officer to the St. Mary's Hospital for Sick Children, Plaistow. — **Hobhouse,** M.D.Oxon., M.R.C.P., appointed Physician to the Alexandra Hospital for Sick Children, Brighton. **O. H. Hudson,** L.R.C.P.I., M.R.C.S.Eng., appointed Medical Officer of Health to the Dronfield Urban District. **T. D. Kirk,** M.D., M.Ch.R.U.I., appointed Medical Officer of Health for the Montgomery Urban District. **Cuthbert Lockyer,** M.D., B.S., M.R.C.P.Lond., F.R.C.S.Eng., appointed Honorary Gynæcologist to St. Mary's Hospital, Plaistow. **Robina McGregor,** M.B., Ch.B.Edin., appointed House-Surgeon to the Victoria Hospital for Sick Children, Hull. **W. Leslie Mackenzie,** M.D.Aberd., D.P.H., appointed Medical Inspector to the Local Government Board for Scotland. **Ernest J. Manning,** M.R.C.S.Eng., L.R.C.P.Lond., appointed Junior Assistant House-Surgeon to the Royal Devon and Exeter Hospital. **C. M. Mitchell,** M.D., appointed District Medical Officer of the Oakham Union. **J. B. K. Robb,** M.A., M.D.Aberd., appointed District Medical Officer of the Burnley Union. **Leonard Robinson,** M.D.Edin., M.D.Paris, appointed Visiting Physician to the Hertford British Hospital, Paris. **Gerald Sheahan,** L.R.C.P. & S., appointed House-Surgeon to the Royal Southern Hospital, Liverpool. **G. W. Sidebotham,** M.R.C.S.Eng., appointed Certifying Factory Surgeon for the Broughton Astley District of Leicestershire. **Alexander Simpson,** M.A., M.D.Aberd., appointed Medical Superintendent of the New Lancashire County Asylum, Winwick, Newton-le-Willows. **C. H. Thompson,** M.D., M.R.C.P., appointed Clinical Assistant to the Chelsea Hospital for Women. **Arthur E. Whitehead,** L.R.C.S., L.R.C.P.Edin., appointed Assistant Resident Medical Officer to St. Mary's Hospital for Sick Children, Plaistow.

